# Historical and contemporary factors that influenced the emergence and continuity of WANEL: Lessons for sustainable HPSR network formation in LMICs

**DOI:** 10.4314/gmj.v56i3s.4

**Published:** 2022-09

**Authors:** Selina Defor, Uta Lehmann, Sue Godt, Issiaka Sombie, Irene A Agyepong

**Affiliations:** 1 School of Public Health, University of the Western Cape, Cape Town, South Africa; 2 Ghana Health Service, Dodowa Health Research Centre; 3 Retired, independent consultant, Canada; 4 West African Health Organization, Bobo-Dioulasso, Burkina Faso; 5 Public Health Faculty, Ghana College of Physicians and Surgeons

**Keywords:** HPSR, West Africa, WANEL, Emerging Leaders, ECOWAS, Health Systems, Network Emergence, Network Formation

## Abstract

**Objectives:**

To explore historical and contemporary factors and processes that influenced the emergence of WANEL and analyse how the formation process has influenced the network's continued existence and sustainability and lesson for sub-regional health policy and systems research (HPSR) networking in Low -and -Middle -Income Countries (LMICs)

**Design:**

Qualitative explanatory case study which used process tracing to chart the formation and development of WANEL.

**Methods:**

Data was obtained through document reviews, semi-structured interviews, group discussions, and participant observation. Data was analysed using thematic content analysis.

**Results:**

The emergence of WANEL was made possible by several factors, including support from a network of senior HPSR champions and institutions across West Africa; sustained funding from IDRC Canada, a reputable funder with a track record in supporting research capacity development in LMICs; learning and networking opportunities provided by CHEPSAA Emerging Leaders and the Institute of Tropical Medicine Antwerp Emerging Voices for Global Health initiative. Its formation followed a mix of emergent and engineered processes.

**Conclusion:**

WANEL is the first and currently the only sub-regional network for early and mid-career health policy and systems researchers and practitioners in West Africa. To ensure its long-term sustainability, the network needs to put in place mechanisms to constantly attract and develop the next generation of early and mid-career researchers, maintain links with senior researchers, strengthen its capacity for coordination and facilitation, and develop a plan for its long-term financial sustainability.

**Funding:**

The study is funded by IDRC Canada Project 108237-001: Popularly known as the Consortium for Mothers, Newborn, Children, Adolescents and Health Policy and Systems strengthening in West and Central Africa. (COM-CAHPSS)

## Introduction

Low -and -Middle -Income Countries (LMICs) constitute about three-quarters of the world's population living with the largest burden of diseases. 1 Their health systems often struggle to scale up proven effective health interventions. The multi-disciplinary field of Health Policy and Systems Research (HPSR).^2^–^5^ has the potential to support LMIC health systems to better fulfil their mandate to assure the health of populations.^6^–^10^ However, persistent capacity gaps continue to limit the generation and use of HPSR in LMIC.^10^,^11^

The Economic Community of West African States (ECOWAS) comprises 15 LMICs with an estimated population of about 350 million. The Ebola epidemic starkly exposed the vulnerabilities of health systems in some of the poorest countries in this sub-region.^12^,^13^ The region's HPSR output though growing, remains limited.^11^, ^15^ Scientific collaboration and cross-disciplinary engagement between institutions and individuals across countries have been hampered by Anglophone-Francophone linguistic barriers.

The sub-region has a very young population 14, which represents a resource that could generate a brain gain^16^–^18^ and contribute to development and health outcome improvement^19^ if adequately harnessed and supported.

The West African Network of Emerging Leaders in Health Policy and Systems (WANEL) initiative emerged in the ECOWAS sub-region in 2015 to support early and mid-career researchers and practitioners in the sub-region to generate and promote HPSR for health outcome improvement. The network provides space for peer-to-peer exchange and learning and facilitates access to research training and leadership opportunities while connecting members to relevant global networks. From an embryonic network of 29 founding members in June 2015, WANEL currently has over 100 members from 13 out of the 15 member states of the ECOWAS. Members are from diverse HSPR-related disciplinary and professional backgrounds, including health policy and systems practitioners, policy advocates, researchers, and policymakers. WANEL provides an active learning environment for the acquisition, consolidation, and transfer of HPSR competencies among early and mid-career HPSR researchers and research users. The emergence and growth of WANEL have been actively supported by strategic collaborative engagements with the West African Health Organization (WAHO) and senior researchers in the ECOWAS sub-region and globally. The network is currently legally incorporated as a professional association in Ghana, where its secretariat is located.

A considerable knowledge gap exists regarding how networks emerge, evolve, and are sustained. Some recent studies have described how investments in research training have contributed to the development of networks and social capital 20,21 for the development of the field of health policy and systems research.^22^ This study is a subset of a larger study that tries to fill this knowledge gap with its overall research question: “how did WANEL emerge and how and why does the network function to contribute to HPSR evidence generation and use capacity among emerging researchers in West Africa.” It specifically explores historical and contemporary factors and processes that have influenced the emergence of WANEL and analyses how the formation process has influenced the network's continued existence. Subsequent papers, which are part of the same study, will explore the network's structural characteristics and operational processes to determine its effectiveness, long-term sustainability chances and lessons for sub-regional HPSR networking in LMIC.

## Methods

### Study design

The study design was a qualitative explanatory case study using process tracing^23^,^24^ to chart the formation and development of WANEL. Document review, participant observation and semi-structured interviews were used to collect data. Documents reviewed included WANEL and COMCAHPSS reports and records as well as material from organizations and institutions whose direct and indirect contributions provided the impetus for the emergence of WANEL, such as WAHO, International Development Research Centre (IDRC) Canada, ECOWAS Commission, Consortium for Health Policy and Systems Analysis in Africa (CHEPSAA), Council on Health Research for Development (COHRED), and the Emerging voices (EV) secretariat. These documents included blog posts, concept notes, email correspondence, grant proposals, agenda and minutes of meetings, reports, institutional websites, video clips, notes from participant observation and transcripts of informal discussions.

Semi-structured interviews sought to elicit the beliefs, values and expectations of the network's founding members. A total of seven interviews were conducted with founding members from Ghana (2), Benin (2), Burkina Faso (1), Nigeria (1) and Senegal (1), plus a group discussion with three founding members from Cote d'Ivoire (1) and Ghana (2). Additionally, interviews also took place with three key stakeholders from WAHO and IDRC. The observation notes of the first author who worked as a project administrator at the secretariat of the West and Central African Health Policy, Systems and Maternal, Newborn, Child and Adolescent Health partnership (better known as the *Consortium for Mothers, Children, Adolescents, and Health Policy and Systems Strengthening (COMCAHPSS*), provided complementary primary data for the study.

### Data Analysis

Document analysis was used to map the historical sequence of events and to identify key actors and other stakeholders. interview transcripts, information extracted from desk review, and observation notes were analysed manually for themes, commonalities and contrasts. A theoretical /conceptual framework was used to guide data collection and analysis. Additional to the themes in the framework, emergent themes not captured by the framework were explored.

### Theoretical / Conceptual framework

**Figure 1:**
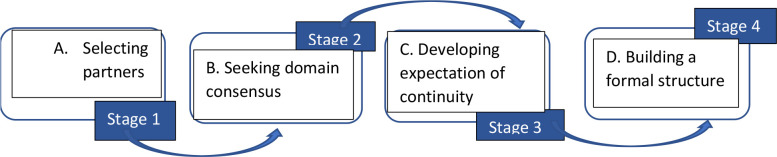
Network formation framework

The larger study of which this study is a part draws on the network effectiveness evaluation framework developed by Adelle et al 25 and Wind's network sustainability factors.^26^ Adelle's framework proposes a combination of factors from three key network elements i.e. network structure, process and outcomes that are required for effective network functioning. This study focuses predominantly on questions related to process, which is just one of the three elements in Adelle's framework. To focus on process, the theoretical framework for this study draws on the network formation process and development theories and frameworks from Doz et al. 27, Ring et al 28 and UNICEF.29 Both Doz et al., and Ring et al. observed that formal network formation processes follow either emergent or engineered pathway depending on the initial conditions at play. Conditions that lead to an emergent pathway are the perception of environmental inter-dependence or the recognition of similar interests among the potential network participants, driven by factors such as pre-existing social relations and other shared participant characteristics. Conditions that lead to an engineered pathway are the existence of a triggering entity to initiate, orchestrate and drive the formation of the network. Regardless of whether a formation pathway is emergent or engineered, interconnected formation activities or sub-processes are observed. These are partner selection which includes all the mechanisms that are employed to identify, contact, and select potential network participants; consensus building on the network's domain which refers to the negotiation processes and agreements on the network's vision, mission, goals and objectives; participant's expectation of continuity which consists of the network's value proposition and all actions aimed at eliciting commitment from the network members and finally designing a formal network structure which refers to the mechanisms that are put in place to create structures that facilitate member interaction, communication, cooperation, decision-making and conflict resolution procedures. The UNICEF four-stage network development framework is similar in its description of network formation activities. It describes an activation stage where network participants and stakeholders are identified; a framing stage where network rules are aligned with values, standards and perceptions of participants; a mobilising stage to generate and build commitment for the network and its purpose; and a synthesizing stage to blend and improve conditions for productive interaction among participants.^29^ These two frameworks were combined to produce figure 1 as the conceptual framework for this study.

### Ethical considerations

Ethical approval for the study was secured from the University of the Western Cape (Ref No. BM20/4/16) and the Ghana Health Service-Ethics Review Committee (Ref No. GHS-ERC015/03/20).Informed consent was obtained from all the participants.

## Results

### Contextual factors

**Figure 2:**
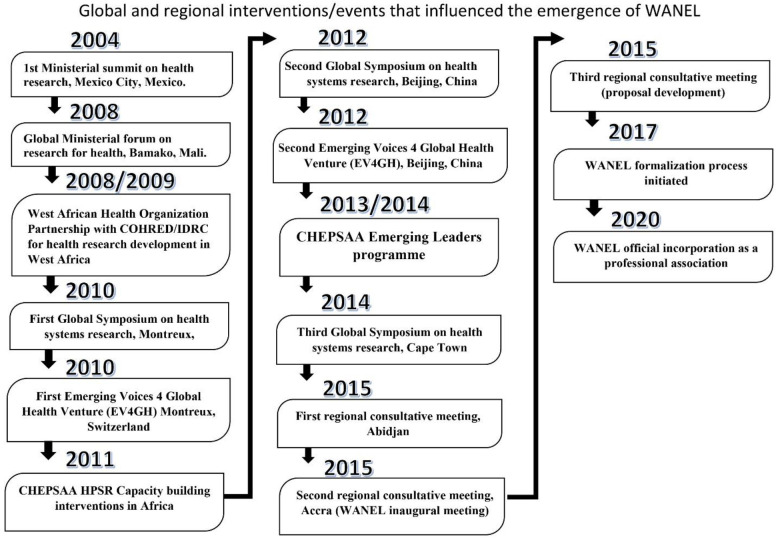
Selected global and regional events and interventions that have influenced the emergence of WANEL

The emergence of WANEL has been influenced by several historical and contemporary global and regional events and interventions as summarized in figure 2 and elaborated in the accompanying text. The health research promotion agenda of the West Africa Health Organization (WAHO)

In 2007, ECOWAS introduced institutional reforms which transformed the ECOWAS Executive Secretariat into a Commission, with the view to adapting to the international environment and strengthening the institution towards achieving its regional integration goal. 30 As part of these reforms, WAHO in 2008, through its Directorate of Research and Health Management Information Systems, put in place mechanisms to work with member states to strengthen health research systems in the sub-region.^31^ WAHO embedded a research promotion agenda in its 2009–2013 strategic plan^32^ and set up a research support fund to provide technical and financial support to the Member States to develop national health research governance structures and policy frameworks. WAHO provided funding for the implementation of approximately 25 operational research projects and trained more than 400 people in research methodology, ethics, fundraising, and research information management. The introduction of WAHO scholarships for health research training benefited several master's and PhD students across the region.^33^

WAHO facilitated the establishment of a regional network of research institutions, the creation of a regional scientific journal, and a two-yearly regional scientific congress. It also engaged with global bilateral and multi-lateral funders such as IDRC, COHRED, the Wellcome Trust, and USAID to support its strategy.

### IDRC HPSR priorities in West Africa

IDRC Canada supported health systems research in LMICs through its Governance for Equity in Health Systems (GEHS) programme.^34^ In 2002, the GEHS programme started supporting the strengthening of local research capacity to analyse, evaluate, and improve performance. 36 West Africa was prioritized due to the fragmentation of the research community and the persistent weak capacity for health systems research. IDRC adopted a new regional strategy for West Africa^34^,^35^, based on which it supported WAHO to implement two foundational programmes. These were the Strengthening research for health system development in West Africa Project (ID 106498), implemented in collaboration with COHRED in Guinea Bissau, Liberia, Mali and Sierra Leone; and the West African Initiative for capacity building through health systems research (ID 106948) aimed at setting the foundation for the production and utilization of rigorous and relevant evidence to strengthen equitable and sustainable health systems. The implementation of these projects led to the establishment of a Regional Consultative Committee (RCC) of senior HPSR researchers, teachers, and leaders in the West African sub-region. In 2015 IDRC provided a small grant through the University of Ghana School of Public Health (UG-SPH) led by the then Chair of Health Systems Global, a West African and a member of the RCC, and supported by WAHO for a planning process to enable deeper consultations across the ECOWAS sub-region to inform the development of a program to strengthen HPSR generation and use capacity in the ECOWAS sub-region. The first regional consultative meeting under the planning grant was held in Abidjan, Cote D'Ivoire, from 26^th^-27^th^ February 2015 as part of a WAHO sub-regional forum with the participation and engagement of the RCC. A second sub-regional consultation was held in Accra in June 2015 and focused on bringing together emerging early and mid-career and senior health policy and systems research and practitioners. The ideas from these consultations in 2015 were developed into the program of the 5year West and Central African Partnership for Maternal Newborn Child and Adolescent Health, better known as COMCAHPSS, that was funded by IDRC in 2016 (IDRC Grant # 108237). One of the six objectives of the work packages of this program was to support the establishment of WANEL as a sustainable early and mid-career West African HPSR network.

### Pre-existing networks and informal relationships

The West African alumni network of the Institute of Tropical Medicine's (ITM) Emerging Voices for Global Health (EV4GH)^36^ and the CHEPSAA Emerging Leaders Programme (ELP) meant that there were pre-existing loose networks of early and mid-career HPS researchers and practitioners in West Africa before WANEL. By 2014, the EV4GH programme had trained 31 West African early-career HPSR actors from Benin (4), Burkina Faso (4), Ivory Coast (5), Ghana (3), Nigeria (8), and Senegal (7). Currently, EV4GH is an integral part of HSG and runs concurrently with the biennial Global Symposium on Health Systems Research.

CHEPSAA was a European Union-funded consortium of African and European Universities that sought to increase sustainable African capacity to produce and use health policy and systems research.^37^ The Emerging Leaders Programme (ELP) of CHEPSAA aimed to contribute to building a critical mass of skilled future leaders in HPSR in Africa. The ELP beneficiaries were from CHEPSAA partner organizations in Nigeria, Ghana, Kenya, Tanzania and South Africa. The programme ran over a period of 18months and in West Africa trained 4 beneficiaries from Ghana and 2 from Nigeria.

### Processes

#### Selecting members

The formation of WANEL began with the 2^nd^ regional consultation supported by the IDRC planning grant in June 2015. Participants were a mix of 18 West African EV4GH and ELP alumni network members and 11 other early and mid-career researchers and practitioners in the sub-region who did not belong to any of these networks.

*“I was very pleased when I got invited as a former emerging voice of global health to participate in the early discussions about the development of a network......that will be like a backbone for the development of the of HPSR in West Africa” **(WANEL Executive Board Member, 27/10/2020)***

The EV4GH and ELP participants were very confident that they had acquired enough skills and competencies as emerging HPS researchers and could contribute to the development of the field in West Africa.

*“I must also say that, one of the things that gave me an upper edge was the fact that I was already a member of the EV4GH......., I think they were looking for people with this kind of background to come together and see how we can push that WANEL agenda forward” **(WANEL Executive Board Member, 8/10/2020**)*

The meeting was attended by participants from nine out of the fifteen ECOWAS countries with disciplinary backgrounds as diverse as Medical science (7), Health Economics (6), Medical Anthropology (3), Sociology/Social Anthropology (5), Public Health (4), Population Studies (1), Political Science (1), and Epidemiology (1) and varying educational levels: 12 PhD students, 7 post-doctoral researchers and 10 Master's degree holders. Senior HPSR researchers in the sub-region, including members of the RCC, also attended this meeting. The lack of linguistic capacity limited access to and engagement with Lusophone West Africa. Francophone West Africa was well represented, with participants from Burkina Faso, Ivory Coast, Mali, Senegal, and Niger.

A special “coaching session with emerita leaders” enabled the emerging leaders to draw from the wealth of experience and knowledge of some distinguished senior and retired leaders who played critical roles in developing the field of HPSR in Ghana. Lessons ranged from how the emerging leaders could develop an open mind that welcomed and respected diverse disciplinary contributions for the development of the field of HPSR, and also emerge with the right mentorship mindset in a context where “mentors” have the tendency to suppress rather than develop talents for fear of being overtaken by mentees.

### Seeking domain consensus

The 2015 consultation, though aimed at eliciting ideas on what was needed to build up the field in the region, also provided the opportunity to discuss the focus of activities of what became WANEL. The meeting agenda followed a participatory process and reflected the HPSR field-building priorities of the participants. Secondly, given the wide pre-meeting online consultative activities, it was possible to assign facilitation roles that aligned with the interest and capabilities of participants. Outcomes from the two-day discussion informed the definition of the WANEL mission and vision (see table 1). The ideas from this meeting were incorporated into the proposal to IDRC for HSPR field building in West Africa to include support for the development and establishment of WANEL.

**Table 1:** WANEL mission and vision

**Mission**	**To foster a bilingual, cross-country network of practitioners and researchers based in West Africa, and increase the** **number of junior and mid-level West Africa-based HPS practitioners and researchers with the competencies required** **for HPS**
**Vision**	A new generation of regional HPS research and practice leaders prepared, in turn, to mentor a future generation of HPS leaders in West Africa
**Values/Principles**	Mutual respect Integrity Trust Confidence in leadership Teamwork/ team spirit Professionalism Continuous learning Peer-support system

**Figure 3:**
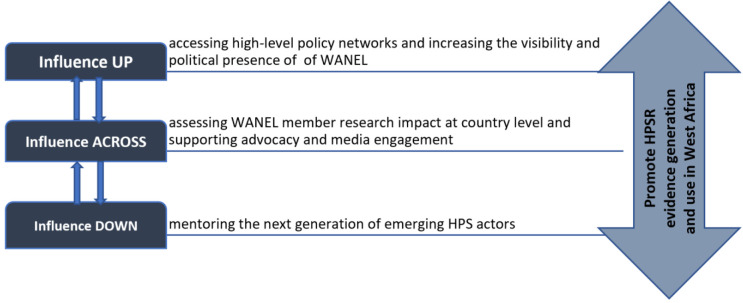
WANEL Implementation strategy as a research policy network

Once funding was successfully obtained, the next WANEL sub-regional meeting was held from February 6^th^-7^th^ 2017 in Niamey, Niger. It provided the opportunity for members to start the development of a strategic plan and consolidate agreement on the values and principles to guide members as they interacted with each other and with other stakeholders. At this point, the founding members had begun to explore possibilities of moving the network into more generative spheres. Thus, to ensure a maximum representation of the perspectives of the broad array of HPSR constituencies, the core organizing team used snow balling procedures to identify and bring on board media journalists, Civil Society Organization and private sector actors from both Francophone and Anglophone West Africa. At this meeting, members also agreed on an implementation strategy involving three axes of influence (see figure3) to realize the network's objectives and vision of being a relevant sub regional research policy network.

### Developing expectations of continuity

In pursuit of its objectives, WANEL carried out an interest and skill mapping exercise to identify both the resources available to the network and the expectations of its members to inform the planning of the network's activities. The outcome informed the setting up of four Thematic Working Groups (TWG) on teaching and learning health policy and systems research, generating and translating evidence into action, supporting health systems strengthening initiatives and advocacy and communication. Currently, about 98% of WANEL members belong to at least one TWG. In line with its vision to mentor the next generation of HPSR actors and also link members to similar global networks, WANEL inaugurated the fifth TWG in 2019, intending to support applications of researchers from Francophone West Africa to the EV4GH and reduce the underrepresentation of Francophone researchers in the HSG community.

Furthermore, joint activities and envisaged collaborative engagements with the ‘Alliance for HPSR development in West Africa and its partners inspired more confidence in the network's future orientation and, consequently, the enhanced expectation of its continuity.

'*There were two main issues which emerged from the Accra meeting which we can address here: one was access to regional HPS short course training, and mentorship opportunities. These will be met as part of the broader partnership activities over the next several years.* (**Network coordinators email ......26/8/2016**)

With a dedicated budget for the development of WANEL, COMCAHPSS supported a total of 44 early and mid-career researchers to attend the fourth and fifth HSG Global Symposia in 2016 and 2018, respectively as well as the Fifth AfHEA Biennial Scientific Conference in 2019. COMCAHPSS also mobilised senior researchers across West Africa to provide abstract review support for WANEL members ahead of the Fourth Global Symposium. WAHO's satellite session at the fourth Global Symposium under the theme ‘*Supporting HPS Evidence Generation and Use Capacity in West Africa......’* profiled WANEL members and the network's potential to support the development of the field in West Africa. WAHO and its partners also involved WANEL members as partners in the implementation of an IDRC-funded research project in 2019 to monitor progress towards the achievement of the health-related SDGs in West Africa.

### Building a formal network structure

In the first five years, the COMCAHPSS secretariat hosted and provided administrative and logistical support to the network's operations. At the second WANEL meeting in Niamey, members dedicated a session to discussing how WANEL should be structured from a governance perspective. Members agreed to have “activity-oriented” governance, which was lean yet encouraged regular dissemination and information sharing. It was agreed to maintain a two-tier system of governance which consisted of regional and country-level structures. The network adopted a decentralised participant-governed governance structure that divided governance responsibilities among subsets of network members across member countries and linguistic divides but continued to depend on the COMCAHPSS secretariat for administrative support. The formalisation process was interrupted by the departure of the first Chair of the network. The vice Chair stepped in as Chair, and a new vice chair was appointed. WANEL put in place a governance structure comprising the WANEL Governance Committee, which included the Chair, Co-Chair, Secretary, and Network Administrator as the highest decision-making body. This was accompanied by the WANEL Executive Board, which is comprised of WANEL country coordinators and the leaders of the five TWG. The network depended to a large extent on the voluntary energies and time of members, and this made it more and more difficult to follow through with the development of the key operational guidelines to guide the network and its operations, given the extensive time and effort needed. The network's constitution for instance, has been pending since 2018 due to time constraints from the drafting committee.

*... ‘Thanks for your suggestion. I also agree with Prof's observations, but it will require an extensive review. At this time of the year, I don't know how many of us that will be available to give time to it. .......... I'm leading a start-up on cancer financing organization with ambitious KPIs, and my schedule is crazy. I'm afraid I may not be available anytime soon to give the document the desire attention'*. (**E-mail response from the chair of the Constitution drafting committee....... 18/01/2018**)

## Discussion

In this paper, we have explored and analysed the processes of emergence and continued existence of WANEL and the historical and contemporary contextual factors that have influenced this. Creech and Ramji suggest that the life of a network is characterised by four dynamic phases of start-up, growth, decline and long-term sustainability.^38^ Across this cycle, networks can experience failures at the beginning or later in the life of the cycle. 39 Network formation can be fraught with challenges that can either hinder the start- up and lead to a ‘blocked network’ or hinder the further development of the network after the successful start- up and lead to a ‘dormant network.^40^

Over the last seven years, WANEL has emerged, survived and continued to grow despite the resource-constrained context of West Africa. Though Doz et al., observed that network formation processes can follow either emergent or engineered formation pathways, it appears that WANEL has followed the third option of a mix of both pathways. Its development was, to some extent, engineered by the support and encouragement of senior HPSR researchers in West Africa involved in the Regional Consultative Committee (RCC), the funding provided by IDRC, and the consistent support of WAHO. However, WANEL's development has also followed an emergent pathway driven by the recognition of similar interests and the need to work together among early and mid-career researchers and practitioners in West Africa who had already been networked through their engagement in the ITM emerging voices (EV) initiative and the CHEPSAA emerging leaders (EL) initiatives. The mutual interests and pre-existing social ties and learning from engagement in these global networks among the founding members were as critical for forming the network.

Ownership is a crucial element for the sustainability of networks. The extent to which a network is owned is revealed typically in members' active engagement within the network. According to Bernard, ownership goes beyond simply performing the business of the network but requires taking responsibility for ensuring that that business remain important, beneficial and well implemented.^41^ In addition to activating the network based on the expressed needs and priorities of members, varied consensus-building processes, including crowdsourcing 42 have ensured collective buy-in for the network's purpose and direction.

It's been further argued that a network's ownership is improved when members are involved in the initial visioning for the network and when they clearly understand its purpose. Thus, the absence of a shared interpretation of a situation in a network (framing) can hinder action steps (mobilising) and result in the failure of the network.^43^ In addition to facilitating domain consensus building, studies have shown that shared interests among network participants causes participants to make economic, social and psychological investments that encourage continued interaction and involvement in the network.^44^ The majority of WANEL founding members have seen remarkable growth in their respective career paths and may be considered to have ‘emerged’ but are still actively involved in WANEL activities. A member of the WANEL Executive Board described WANEL as a ‘baby’ that they love, are happy to watch grow and willing to contribute anything to support its further development.

Bernard further argues that the capacity of individuals to engage in and sustain a network is conditioned by the kinds of support available to provide them a minimum of recognition, legitimation, and links to practice.^41^ WANEL has been consistently supported by reputable regional HPSR champions, the first chair of Health Systems Global, the acting Director of the department of research and public health of WAHO and successive Director Generals (DGs) of the WAHO and IDRC since it emerged in 2015.

Having an institutional home is also an important part of network development and sustainability. Networks stand a better chance of securing funding when they are housed within an institutional home of good repute because they become a more recognisable entity than a loose set of relations among researchers and other stakeholders. The network also benefits from the host institution's visibility, credibility and other administrative services.^27^ Since 2016, the Dodowa Health Research Centre of the Ghana Health Service has provided a welcoming home in an established public sector institution with strong institutional governance systems.

A network's ongoing relevance to both the members and the context is another important sustainability factor. Networks are generally more sustainable when they create solidarity around a shared purpose and allow members to work together on common tasks. 41

It has also been argued that networks which undertake and facilitate collaborative projects are more likely to remain relevant to their members and, consequently, more sustainable than networks that only share information.^27^ The ongoing WANEL collaborative research project being implemented across West Africa with financial support from IDRC in 2020 has increased interactivity within the network and allowed the extension of the Network's activity to Lusophone West Africa. Studies have, however, confirmed the inadequacy of voluntary time in effectively supporting a network's operations once it grows out of its pioneering phase and begins to involve a broader range of members.^45^, ^26^, ^29^ This raises concerns about the long-term sustainability of WANEL, which will be explored further in upcoming papers.

## Conclusion

WANEL is the first and currently remains the only early and mid-career or emerging researcher and practitioner sub-regional network for HPSR in West Africa. The network has brought together researchers from diverse disciplinary backgrounds and HPSR evidence users to support the development of the field of HPSR in West Africa. This has been possible because of historical and contemporary events that have provided a foundation for the effort, such as the growth of HPSR as a field and the related emergence of programs like the Emerging Voices, Emerging Leaders, CHEPSAA and Health Systems Global (HSG). The commitment of early and mid-career researchers in West Africa to be part of this network; the support of senior career researchers and institutions in West Africa like WAHO to nurture the effort; and the ability to attract some funding support. The links to global networks such as the Emerging Voices and Health Systems Global are also critical. There are threats to the long-term sustainability of WANEL. A network focused on early and mid-career researchers must put in place mechanisms to constantly attract and retain the next generation of such researchers while maintaining links with senior researchers as coaches and mentors. It is also critical for the network to strengthen its capacity for coordination, project management and administration and put in place realistic mechanisms to guarantee the network's long-term financial sustainability.
